# A toolkit for haptic force feedback in a telerobotic ultrasound system

**DOI:** 10.1186/s13104-021-05806-2

**Published:** 2021-10-24

**Authors:** Reza Fotouhi, Atieh Najafi Semnani, QianWei Zhang, Scott J. Adams, Haron Obaid

**Affiliations:** 1 Department of Mechanical Engineering, University of Saskatchewan, 57 Campus Drive, Saskatoon, SK S7N 5A9 Canada; 2 Department of Medical Imaging, University of Saskatchewan, Saskatoon, SK Canada

**Keywords:** Haptic simulator, Gilbert–Johnson–Keerthi algorithm, Ultrasound, Tele-sonography

## Abstract

**Objective:**

To develop a collision engine (haptic force feedback simulator) compatible with a 5-degrees-of-freedom (DOF) haptic wand. This has broad applications such as telerobotic ultrasound systems. Integrating force feedback into systems is critical to optimize remote scanning. A collision engine compatible with a 5-DOF haptic wand was developed based on the Gilbert–Johnson–Keerthi algorithm. The collision engine calculated force during collision between the wand and a virtual object based on code developed using MATLAB. A proportional force was subsequently returned to a user via the haptic wand, thereby simulating the collision force for the user. Three experiments were conducted to assess the accuracy of the collision engine on curved and flat surfaces.

**Results:**

The average errors in calculation of distances between the wand and virtual object were 2.1 cm, 3.4 cm, and 4.2 cm for the model of the human hand, cylinder, and cuboid, respectively. The collision engine accurately simulated forces on a flat surface, though was less accurate on curved surfaces. Future work will incorporate haptic force feedback into a telerobotic ultrasound system. The haptic force simulator presented here may also be used in the development of ultrasound simulators for training and education.

## Introduction

Patients in remote and rural communities often have limited access to ultrasound imaging due to the lack of specialized sonographers in these communities. These challenges could be tackled with introducing a telerobotic ultrasound system that would allow specialists to remotely operate and control an ultrasound probe and remotely perform ultrasound scanning [1–3].

Most telerobotic ultrasound systems currently lack feedback of applied force on the patient during the examination. Haptic force feedback simulators is to replicate, physical interaction between an ultrasound probe and patient body to give the operator the opportunity to adjust the forces applied during telerobotic scanning [4, 5].

To support integration of haptic force feedback during interaction with a remote object or a virtual object, a new software is required. Many haptic packages which are currently available are proprietary rather than open source. Open source packages, such as V-COLLIDE and SOLID, operate on Linux and are not compatible with haptic devices, such as the 5-DOF haptic wand developed by Quanser which is used in this study [6].

To address this limitation, the objective of this study was to develop a collision engine (haptic force feedback simulator) compatible with a 5-DOF haptic wand and a Windows operating system. When the 5-DOF haptic wand collides with a virtual object, a force is sensed by a user via the haptic wand, providing force feedback. In this study we assess the performance of this haptic simulator on flat and curved surfaces of three different virtual objects.

## Literature review

### Telerobotic ultrasound systems

A commercially available telerobotic ultrasound system is MELODY, developed by AdEchoTech [7–9]. The MELODY system currently does not allow the sonographer to control the amount of force applied by the ultrasound probe (head) and could benefit from haptic technology. *Haptics* is defined as “touch interactions that occur for the purpose of perception and manipulation of objects” [10–12]. Haptic rendering is a functionality that adds certain haptic properties to an object to give it a realistic feel [11].

### Haptic rendering-collision detection

Collision detection algorithms detect collisions between objects and an avatar in a virtual environment [4]. In order to detect a collision, the position of the end-effector (avatar) in the virtual environment must be determined. If the avatar is in free space and not colliding or touching a virtual object, then the calculated contact forces on the interface will be zero. However, if the avatar is touching a virtual object, forces are felt by the user manipulating the end-effector.

### Haptic rendering

Collision response, or force-response, algorithms compute interaction force between avatars and virtual objects. It calculates an appropriate amount of force to be passed onto the haptic interface device. This force, *F*, is calculated using the following equation:

1 $$ F = k \, x + C \, v $$

Here *k* is stiffness of object, *C* is viscous damping, *x* is penetration depth between the object and avatar (e.g. haptic wand), and *v* is the linear velocity of the avatar.

### Collision detection engine

To develop a virtual reality haptic simulator, a software package (collision detection engine) and a haptic interface are required. The purpose of a collision detection engine is to detect collisions between two objects, calculate the distance between colliding objects, calculate the collision force, and generate an equal force via the haptic device. Commonly used algorithms include the Gilbert–Johnson–Keerthi (GJK) algorithm, bounding volume algorithm, and virtual spring algorithm, among others [10, 13–16].

In this toolkit, the GJK algorithm is utilized. This algorithm is a method to detect collisions between two convex objects. The algorithm is based on calculation of the Minkowski difference between two convex objects and determining whether or not the origin included is different. If the origin is included, the two objects collide with each other and have some points in common [17].

### Haptic interface

Several algorithms have been used for training or simulation using haptic interface [13, 18–21].

### Evaluation of haptic interfaces

Haptic interfaces are devices that enable manual interaction with virtual environments. They are employed for tasks that are usually performed using hands in the real world [10, 19, 22]. The haptic device used for this research is a 5-DOF haptic wand. The interface has five degrees of freedom, consisting of three degrees of translation and two degrees of rotation (roll and yaw).

## Main text

### Materials and methods

The collision engine which is developed in this study is based on collision detection using the GJK algorithm [23]. To calculate the approximate amount of force during the collision of the lower tip of a haptic wand and a virtual object, we developed MATLAB code which calculates the force based on Eq. (1) [12]. An overview of the methodology is shown in Figure 4 and described in further detail as follows:

1. 3D objects

Three 3D objects were imported into SolidWorks and saved in STL (stereolithography) format. To compare results between flat and curved surfaces of objects imported into the collision engine, STL files of the following objects were imported into MATLAB and Simulink (see Figure 5):

- A human hand reconstructed from a CT scan [24];
- A cylinder with a 0.025 m radius and a 0.1 m height; and
- A cuboid measuring 0.1 × 0.1 × 0.2 m^3^.

2. Conversion from STL to X3D format

Using Blender (v. 2.49), we converted the STL files to X3D format, which is the required format for Quanser 3D Viewer. The converted human hand model in X3D format is shown in Figure 6. Considering that visualization and force calculation are two parallel and separate processes (Figure 4), a transformation matrix between MATLAB and Blender coordinate systems was used to calculate applied forces from object positions.

3. Import into 3D Viewer

The 3D Viewer was used for visualization of an avatar of the wand’s tip and three objects imported into the virtual environment. Objects represented in X3D format were imported into 3D Viewer. The wand and an object in X3D format in virtual environment are shown in Figure 7.

4. Convert STL files to object vertices

**Fig. 1:**
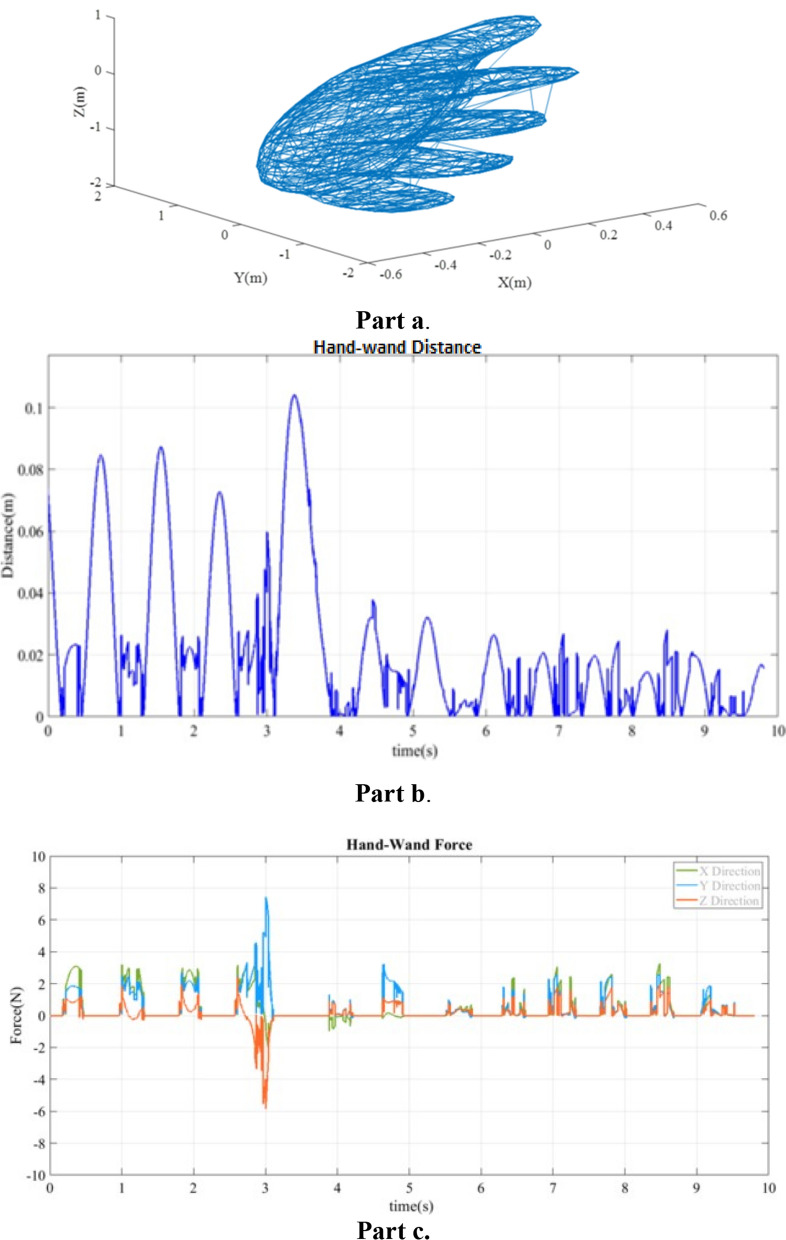
**a** Plot of human hand model vertices; **b** distance sequence for human hand model and wand collision; **c** force sequence for human hand model and wand collision

**Fig. 2:**
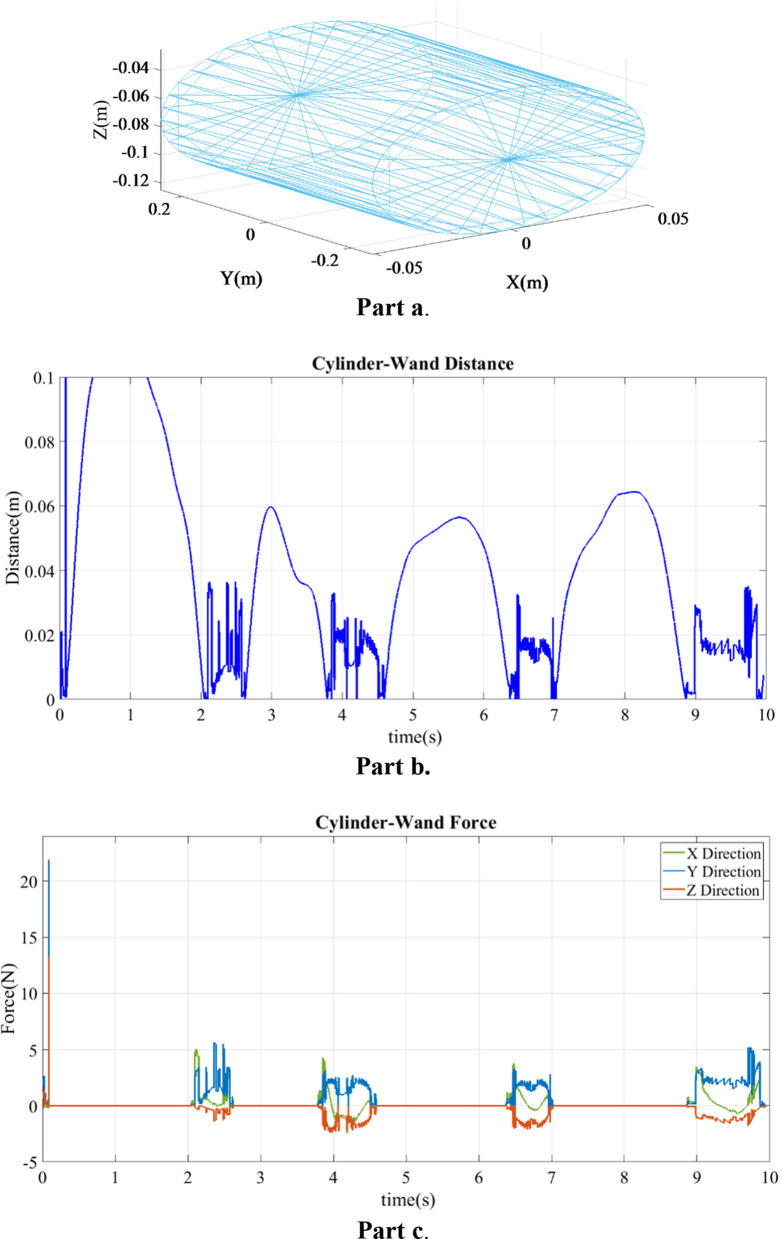
**a** Plot of cylinder model vertices; **b** distance sequence for cylinder model and wand collision; **c** force sequence for cylinder model and wand collision

**Fig. 3:**
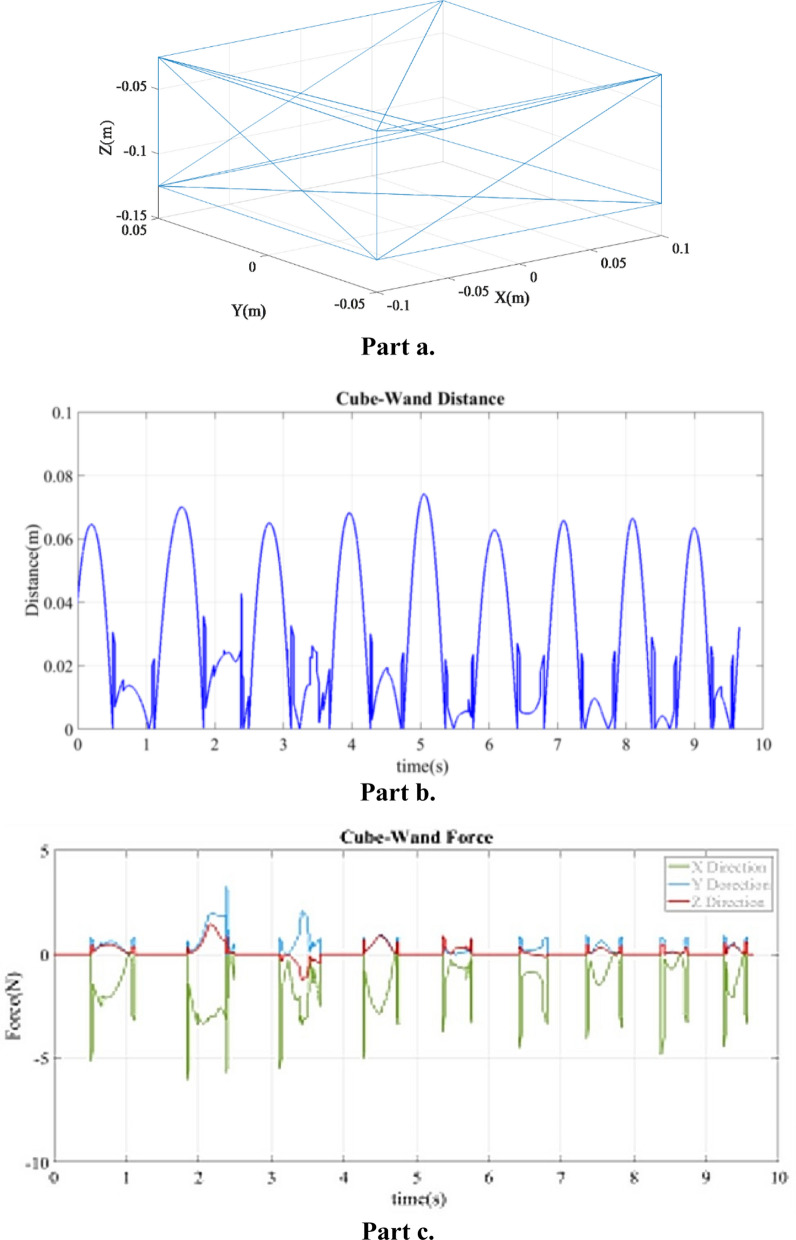
**a** Plot of cube model vertices; **b** distance sequence for cube model and wand collision; **c** force sequence for cube model and wand collision

**Fig. 4:**
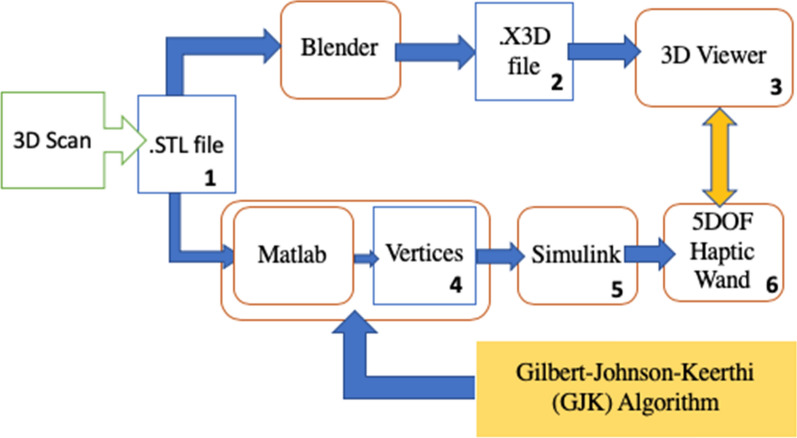
Flowchart of the collision engine. Numbers in boxes refer to steps in Methodology

Using a variation of MATLAB code [25], we converted STL files to object vertices (see Fig. 1a, Fig. 2a, and Fig. 3a). Vertices of an object were used as inputs for the GJK algorithm. These vertices form an n × 3 matrix (where n is the number of vertices, each labeled by 3 coordinates), which define the object in the X, Y, and Z planes. The cuboid, cylinder, and human hand were represented by 36, 456, and 3660 vertices, respectively.

5. GJK algorithm in Simulink and MATLAB

Vertices of the imported objects produced in the previous step, as well as stiffness and damping ratios of each object and real time position vectors of the haptic interface, were used as inputs for the main MATLAB code [25]. For all objects, stiffness in the X, Y and Z directions was manually set to (160, 160, 160) N/m, and damping factors were set to (0.8, 0.8, 0.8) N∙s/m, respectively.

**Fig. 5:**
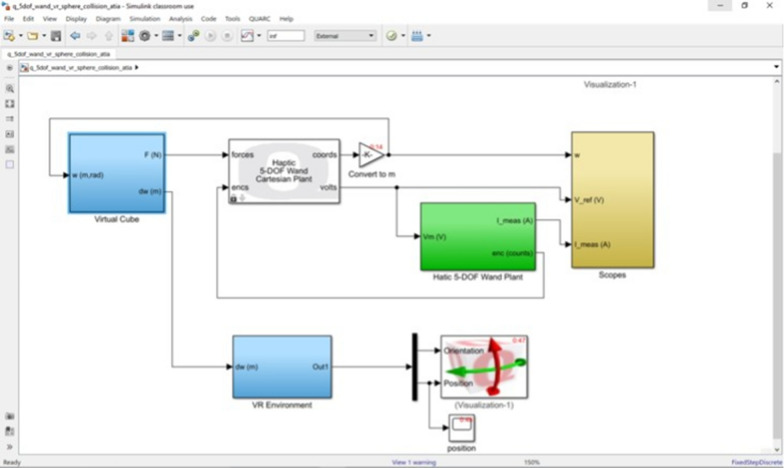
Simulink model for the virtual reality haptic simulator, including QUARC blocks

**Fig. 6:**
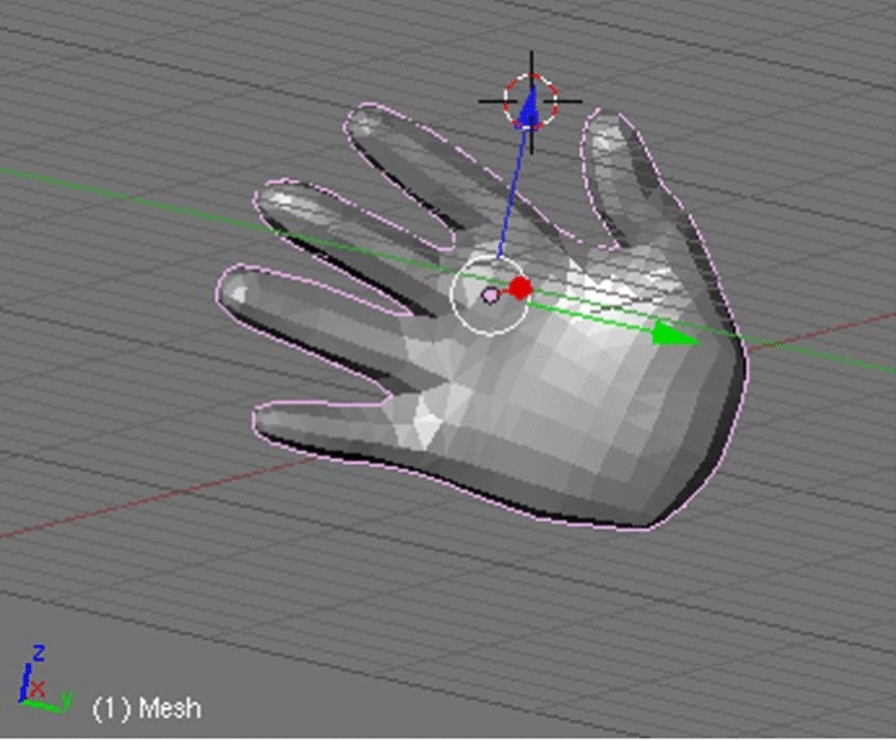
Converting the object from STL to X3D format

**Fig. 7:**
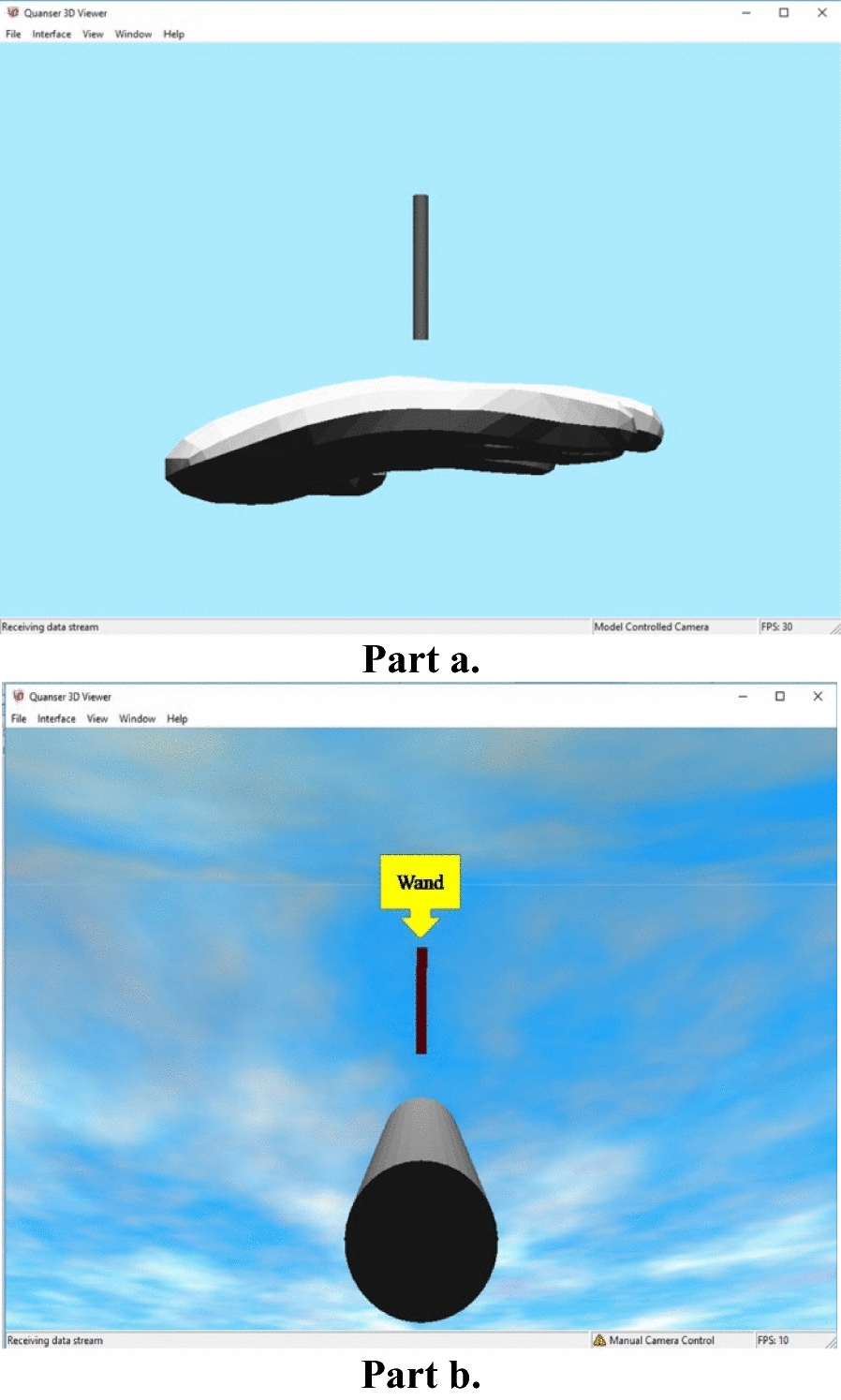
X3D format of the imported models in the Virtual Reality Environment (3D Viewer), with the wand above each object; **a** human hand model, **b** cylinder model

The position of the haptic interface was defined by a 1 × 3 vector. “1” represents a single point (haptic interface), and “3” represents the X, Y, and Z coordinates of this point. The position vector is output of a specific QUARC block called “Haptic 5 DOF Wand Cartesian Plane” (Figure 5).

The center of mass of each object was set to (0, 0, − 0.075). We simplified the wand to be comprised of a single point with one vertex and three degrees of freedom. Initial position of the wand was set at (0, 0, 0).

6. Simulating and generating haptic force feedback

A “CollisionFlag” was deployed so that when a collision was detected, the flag went to “1”; otherwise, it stayed at “0”. If “CollisionFlag” was “1”, the code calculated the force based on Eq. (1). The 5-DOF haptic wand generated force feedback when the wand collided with an object in the virtual environment.

Three virtual experiments were conducted in a 3D-setting to assess the accuracy of the collision engine: dorsal aspect of the human hand, curved surface of a cylinder, and flat surface of a cuboid. In these experiments, over a 10-s period, a user manipulated the haptic wand so that it would collide with the surface of the virtual object. Distance between the wand and the virtual object was recorded and contact forces between the virtual objects and the wand were measured by the collision engine based on Eq. (1) in *X*, *Y* and *Z* directions.

### Results and discussion

#### Experiment with a model of a human hand

Figure 1b shows the distance between the wand and the dorsal surface of the hand in the first virtual experiment in which the wand was collided with a model of a hand over a 10-s period. At collision, the calculated distance between the wand and hand is zero. For this experiment, there is no record of false value for calculated distance from the engine. The simulated contact forces between the hand and wand are presented in Fig. 1c. The average of these forces during contact were 0.998 N, 0.996 N and 0.345 N in *X*, *Y* and *Z* directions, respectively. The average of the forces for all 11 contact periods is given in Table 1.

#### Experiment with a cylinder

Figure 2b shows distance between the wand and curved surface of a cylinder in the second experiment where the wand was collided with the cylinder over a 10-s period. In some instances the engine miscalculated distances, which caused a noisy collision response. These miscalculated distances occurred at 6 instances: 3.8, 4.0, 4.2, 6.5 6.9, and 9.0 s, with an average error of 3.44E-2 m. The simulated contact force between the cylinder and wand are presented in Fig. 2c. The average of these forces during contact were 0.3561 N, 1.711 N and 0.6248 N in *X*, *Y* and *Z* directions, respectively, as shown.

#### Experiment with a cuboid

Figure 3b shows distance between the wand and surface of a cuboid in the third experiment where the wand was collided with the surface of a cuboid. In this experiment, the engine returned a false value for the calculated distance at instance of 2.4 s, which led to a noisy collision response, with an average distance error of 4.176E-2 m. The simulated force during contact between the cuboid and the wand in *X*, *Y* and *Z* directions is shown in Fig. 3c. The average contact forces in *X*, *Y* and *Z* directions were 1*.*62 N, 0*.*477 N, and 0*.*217 N, respectively, as shown. The average of the forces for all 9 contact periods is given in Table 4. By comparing the results of the two experiments, as shown in Table 2, it can be observed that the collision engine can detect the collision between the wand and a flat surface more efficiently than with a curved surface. Reported results are experiments between the haptic wand, manually controlled by an operator, and different virtual objects; the speed of wand motion, and shape of each object affects contact intervals.

To verify the generated force based on Eq. (1) from Simulink, we manually calculated these forces for two random instances at 8.098 s and 8.436 s in the cuboid experiment. The distance between the cuboid and wand at 8.098 s was 6.645E−2 m, with an expected force value of zero at that time. Figure 3c shows that the related force at 8.098 s is equal to zero in *X*, *Y*, and *Z* directions. Figure 3b shows that the distance at instant 8.436 s is − 9.685E−4 m, which means the wand collided with the cuboid. Table 3 shows the wand velocity, distance vector from the wand toward the center of mass of the cuboid, and force value for *X*, *Y*, and *Z* directions at instant 8.436 s. The calculated force based on Eq. (1) at this instant (*F*_*x*_ = *0.3194 N*) is the same as the mean value of the contact forces as shown in Fig. 3c (*F*_*x*_ = *0.3 N*). The mean values of contact forces are reported in Table 4.

It should be noted that contact forces are not constant for each collision; as seen from Eq. (1); forces are dependent on penetration depth (*x*) and wand velocity (v); thus, contact forces may change for each contact; since the damping values used for each virtual objects are small, damping of contact forces are not noticeable.

### Conclusion

In this paper, a collision engine compatible with a 5-DOF haptic wand to simulate haptic force feedback is presented. Through virtual experiments with cuboid, cylinder, and human hand models, we have shown that our approach can work on several surfaces. The collision engine was able to accurately simulate haptic force feedback on a flat surface, though was less accurate on curved surfaces. One of the limitations of the GJK algorithm [16] which was utilized in this research is that it can only detect collisions between convex objects. The haptic force feedback simulator presented here will have applicability in development of ultrasound simulators for training and education, and in incorporating force feedback for telerobotic ultrasound system.

## Limitations

Haptic packages which are utilized in the majority of research works include Phantom Omni and Open Haptics [18]; however, neither are open source. V-COLLIDE and SOLID are two open source software packages commonly implemented as collision engines [12], both on Linux.

V-COLLIDE is an n-body processor used for multiple object collision detection. Once a user assigns the position of objects to V-COLLIDE, the software will return contact status of the objects including collision or any possible contact. Although V-COLLIDE is a powerful tool for dynamic collision detection and recalling the position of objects, this software is unable to calculate distance between objects [26]. SOLID is another collision detection library utilized for multiple three-dimensional polygonal objects going through rigid motion. SOLID is comparable with V-COLLIDE in terms of performance and application, and it determines interface between objects [27]. In comparison with V-COLLIDE and SOLID, the collision engine presented in this paper can be used on a Windows operating system and facilitates measurement of distance between colliding objects.

The proposed algorithm utilized in the collision engine is limited since it can only detect collisions between convex objects. In the future, the convex decomposition method [18] can be incorporated into the engine to remedy this. The program currently does not record the trajectory of the wand, although this feature may be added in the future. To contact from different direction, as shown in above examples, we can rotate them (e.g. cuboid) in virtual setting; because of physical construction of the haptic wand, it has limited range of motion, which is about ± 30° about Z-axis.

## Data Availability

Data are codes available publicly at HF2MS in https://sourceforge.net/projects/hf2ms/files/; and in https://github.com/atiandjf/HF2TS).

## Appendix

See Figs. 4, 5, 6, 7; Tables 1, 2, 3, 4.

**Table 1:** Contact intervals with mean values of measured forces in *X*, *Y*, and *Z* directions, Human Hand Model

Time period (s)	F_x_ (N)	F_y_ (N)	F_z_ (N)
0.2–0.45	2.434	1.542	0.8417
1–1.3	1.799	1.407	0.1984
1.8–2.1	2.204	1.647	0.5779
2.65–3.05	1.191	2.791	− 1.133
3.88–4.25	− 0.2231	0.2434	0.2146
4.6–4.95	0.003	0.1909	0.7829
5.5–5.9	0.3367	0.2689	0.2665
6.3–6.65	0.4804	0.3912	0.2679
6.9–7.3	0.7628	0.6750	0.4729
7.65–8.1	0.8455	0.8792	0.5574
8.35–8.7	1.154	0.9189	0.7470

**Table 2:** Comparison of experiments performed for the flat surface of the cuboid and curved surface of the cylinder

	Number of vertices	Average of distance error (m)	Number of errors	Total collisions
Cuboid	36	4.176E-2	1	18
Cylinder	456	3.44E-2	6	9

**Table 3:** Force calculation at instant 8.436 s, Cuboid

	Wand velocity (m/s)	Distance vector (m)	Force (N)
X	0.2055	− 9.685E−4	0.3194
Y	0.0087	1.967E−4	0.0245
Z	0.0069	1.268E−4	0.0148

**Table 4:** Contact intervals with mean values of measured forces in X, Y, and Z directions, cuboid model

Time period (s)	F_x_ (N)	F_y_ (N)	F_z_ (N)
0.50–1.10	− 2.1	0.3	0.3
1.80–2.40	− 2.9	1.1	0.9
3.10–3.70	− 1.8	0.9	− 0.3
4.25–4.75	− 1.9	0.5	0.5
5.40–5.75	− 0.5	0.1	0.2
6.45–6.80	− 1.1	0.2	0.1
7.40–7.80	− 0.8	0.3	0.2
8.40–8.75	− 0.3	0.1	0.05
9.25–9.60	− 1.4	0.3	0.43

## Abbreviations

3D: 3-Dimensional
DOF: Degrees-of-freedom
GJK: Gilbert–Johnson–Keerthi
QUARC: Real-time control software from Quanser
SOLID: An acronym for the first five object-oriented design principles
STL: Standard Template Library
V-COLLIDE: A collision detection library for large environments (of polygonal objects)
X3D: A royalty-free ISO/IEC standard for declaratively representing 3D computer graphics
